# Influence of chronic exposure to thiamethoxam and chronic bee paralysis virus on winter honey bees

**DOI:** 10.1371/journal.pone.0220703

**Published:** 2019-08-15

**Authors:** Marianne Coulon, Frank Schurr, Anne-Claire Martel, Nicolas Cougoule, Adrien Bégaud, Patrick Mangoni, Gennaro Di Prisco, Anne Dalmon, Cédric Alaux, Magali Ribière-Chabert, Yves Le Conte, Richard Thiéry, Eric Dubois

**Affiliations:** 1 ANSES Sophia Antipolis, Unit of Honey bee Pathology, Sophia Antipolis, France; 2 INRA PACA, UR 406 Abeilles et Environnement, Avignon, France; 3 University of Napoli “Federico II”—Department of Agriculture, Portici, Napoli, Italy; 4 CREA, Council for Agricultural Research and Economics—Research Center for Agriculture and Environment, Bologna, Italy; Universitat Leipzig, GERMANY

## Abstract

Co-exposure to pesticides and viruses is likely to occur in honey bee colonies. Pesticides can be present in pollen, nectar, and persist in stored food (honey and bee bread), and viruses can be highly prevalent in honey bee colonies. Therefore, the present study describes the influence of chronic co-exposure to thiamethoxam and Chronic bee paralysis virus (CBPV) on bee survival, virus loads, expression level of immune and detoxication genes, and pesticide metabolism Experiments were performed on honey bees collected from a winter apiary with reduced viral contaminations. No synergistic effect of co-exposure was observed on bee survival, nor on the ability of bees to metabolise the pesticide into clothianidin. However, we found that co-exposure caused an increase in CBPV loads that reached the viral levels usually found in overt infections. The effect of co-exposure on CBPV replication was associated with down-regulation of vitellogenin and dorsal-1a gene transcription. Nevertheless, the observed effects might be different to those occurring in spring or summer bees, which are more likelyco-exposed to thiamethoxam and CBPV and exhibit a different physiology.

## Introduction

Many studies have provided concurring evidence that several stress factors, acting individually or in combination, are contributing to honey bee colony losses and the decline of wild pollinators: loss of habitats, global warming, decreased availability of food sources, pesticide use in agriculture, and spread of parasites and pathogens [1,2].

Among them, viruses are increasingly being investigated as potential causes of honey bee loss [3–6]. They generally persist in honey bee populations at low levels without clinical signs until the emergence of overt infections. Besides the parasite *Varroa destructor*, which multiplies and transmits large quantities of *Deformed wing virus* (DWV), *Varroa destructor virus -1*, and/or *Acute bee paralysis virus* (ABPV) [7,8] the factors that cause sudden viral outbreaks are poorly understood. At high levels, viral infections usually cause high worker mortality and/or colony losses [9–12].

A factor possibly causing viral outbreaks could be exposure to pesticides. Indeed, some pesticides have been found to impair honey bee immune response, and to be linked to honey bee diseases [13]. Interactive effects between viruses and pesticides of the neonicotinoid family have already been reported. For instance, increased mortality in honey bee larvae with high viral loads of *Black queen cell virus* (BQCV) has been found when these virus-infected bees were co-exposed to sublethal doses of thiacloprid [14]. Significant increases in DWV loads have also been found in honey bees co-exposed to the virus and clothianidin [15]. The authors of this paper also discovered that the honey bees could no longer control the viral replication because the transcription of the *dorsal-1a* gene, an NF-κB effector protein involved in the Toll pathway [16], was inhibited by exposure to clothianidin.

In order to further study the potential role of pesticide exposure on viral loads and more specifically the transition from covert to overt viral infections in honey bees, we analysed the effects of co-exposure to the Chronic bee paralysis virus (CBPV) and the neonicotinoid thiamethoxam. CBPV, which causes chronic bee paralysis [17], is not yet classified, but shows similarities to the *Nodaviridae* and *Tombusviridae* families [18]. Its genome is made up of two segments of single-stranded RNA in a non-enveloped anisometric capsid [19]. This virus has been found to be transmitted horizontally by contact between healthy and sick bees in the hive [20]. Aside from the hairless black body symptom known since Antiquity, tremors, paralysis, and inability to fly are the most frequent and characteristic symptoms [21,22]. As a result, a large amount of dead or paralysed bees can be observed in front of the hives [21,22]. Natural outbreaks occur sporadically, more frequently in the spring and summer [21]. However, confusion with symptoms of pesticide intoxications [23] can occur [21]. CBPV has been shown to have neurotropism, and was observed to be present in the mushroom bodies of infected honey bees, which could cause the specific nervous symptoms [18]. In one study, CBPV was detected in 28% of apiaries in France [24], but only 2% of colonies were reported to show clinical signs of the disease [25]. In Europe, various prevalence rates have been reported [26,27] but never exceeded 28% (in France, in 2004 [24]). In 2015, a one year long survey across Europe showed a prevalence between 0.60 and 0.92% of clinically infected apiaries, leading to increased winter (18.92%) and seasonal (4%) mortalities [28]. CBPV loads found in bees from symptomatic colonies are significantly higher than in bees from asymptomatic colonies (over 10^8^ equivalent genome equivalents per bee) [29,30].

Thiamethoxam is a neonicotinoid insecticide commonly used around the world on oil-seed rape, a crop that is widespread and attractive to honey bees [31,32]. This pesticide has been reported to be present at various concentrations in the honey bee environment, with for example concentrations reaching 13.3 ng/g in nectar from oil-seed rape, and 86 ng/g in pollen from field margin plants [33]. It has also been detected in hive matrices, at a maximum of 20.2 ng/g in honey [34], and 53.3 ng/g in stored pollen [35]. Neonicotinoids bind with high affinity to acetylcholine receptors, altering neuronal signals, which can lead to paralysis and death of the insect [36]. At sublethal doses, it can have negative effects on homing flights in foragers [37], and olfactory memory and learning [38]. Chronic exposure has been shown to damage the brain and gut of Africanized honey bees [39,40]. Thiamethoxam is quickly and readily metabolised into clothianidin, which is also marketed as a neonicotinoid insecticide, also impacting on insect acetylcholine receptors [36]. Clothianidin has notably been found to inhibit the honey bee immune system, which in turn can promote the replication of DWV [15,41].

In order to study the effect of co-exposure to CBPV and thiamethoxam, which is likely to occur in the field but also within colonies through the contamination of hive matrices, we monitored honey bee survival and viral loads in a laboratory experiment, after 10 days of chronic exposure to thiamethoxam (oral exposure) and to CBPV-infected honey bees (contact exposure). We also selected four genes that are part of immune pathways: *vitellogenin*, *dorsal-1a*, *apidaecin* (also involved in the production of antimicrobial peptides) and *prophenoloxidase* (*ppo*) from the melanisation pathway [15,42,43]. Finally, we selected three genes that play a role in detoxication processes: *glutathione-S-transferase 3* (*gstS3*), *catalase* and *cyp6as14* [44,45]. We also investigated whether CBPV infection could impair thiamethoxam metabolisation to clothianidin, by carrying out a kinetic analysis of pesticide levels in exposed honey bees. Because DWV, SBV, and CBPV can be transmitted by consumption of contaminated pollen harvested in the environment by the foragers [46], thus all experiments were performed with winter bees in order to have a better control of initial viral loads and reduce the risk of interference from other viruses to a maximum.

## Materials and methods

### Winter-born honey bees

Experiments were performed with honey bees (*Apis mellifera*) obtained from three colonies previously tested negative for CBPV, ABPV, *Sacbrood virus* (SBV) and DWV, and located at the ANSES Sophia Antipolis laboratory winter apiary [19] in order to maintain their viral status. Colonies in the winter apiary were fed with 50% sucrose syrup prepared in our laboratory from pure sucrose (D^(+)^-sucrose, Acros Organics, Fisher Scientific, USA) and water, and protein paste, also prepared in our laboratory from candy sugar (Apifonda, Südzucker AG, Germany), fructose syrup (Fructoplus, Icko-Apiculture, France), and a commercial mix of supplemental *Saccharomyces cerevisiae*, ascorbic acid, and various proteins and minerals (Apifeed, SINTAL, Italy).

We tested the influence of thiamethoxam and CBPV co-exposure on bee survival, virus loads and physiology (Experiment 1, February 2016), and on pesticide metabolisation (Experiment 2, March 2017). To obtain bees, frames from three colonies, containing late-stage pupae were collected and placed in an incubator overnight at 34°C. Emerging bees were pooled to minimise colony-borne bias and distributed into cages: 30 bees per cage of about 780 cm^3^ [47]. Cages were maintained at 34°C in incubators with a container filled with water at the bottom to avoid dessication, and bees were fed *ad libitum* with one feeder containing 50% sucrose syrup, a second feeder containing 50% sucrose syrup supplemented with 1% protein (Provita’Bee, ATZ Diététics, France), and a third containing crystallised sugar paste. Nine days after emergence, only the 50% sucrose syrup feeders, supplemented with thiamethoxam or not depending on the conditions, were provided to bees.

### Honey bee CBPV exposure

In order to reproduce natural transmission of CBPV, viral exposure was performed by contact between experimental honey bees and previously CBPV-inoculated honey bees. Five-day-old bees were first anesthetized using CO_2_ and then injected through the thorax with 4.0 x 10^4^ genome equivalent copies of purified CBPV strain A-79P (accession numbers: EU122229.1 and EU122230.1), according to the previously described protocol [19]. After four days of incubation, five (Experiment 1) or nine CBPV-infected bees (Experiment 2) (depending on the difficulty of obtaining a high number of bees of the same age for CBPV inoculation) were paint-marked and used to transmit the virus to nine-day-old honey bees (30 healthy honey bees per cage). A preliminary experiment had shown that both proportions of infected bees per cage had comparable effects on survival and viral transmission [48]. The injected bees (marked bees) died within the first three days, but were not removed, to promote transmission of CBPV which remains infectious through physical contact in the cage. Indeed, CBPV is resistant in the environment, including in pollen, faeces, or dead bees [12,20,22,46].

### Honey bee thiamethoxam exposure

To carry out pesticide exposure, a standard solution of thiamethoxam at 100 mg/L (prepared in water) was diluted in 50% sucrose as previously described [48], to obtain final concentrations of 10 μg/L, and 200 μg/L, corresponding to the expected daily doses of 0.25 and 5.0 ng/bee, respectively [48]. These doses were considered to be field-relevant (see introduction).

### Experiment 1: Influence of thiamethoxam and CBPV co-exposure on bee survival, virus loads and physiology

Nine days old bees were exposed to 50% sugar syrup or 50% sugar syrup supplemented with thiamethoxam, according to the following six conditions (n = 4 cages per condition): *i*. Control bees (bees not exposed to CBPV nor to thiamethoxam); *ii*. Bees in contact with CBPV-infected bees; *iii*. Bees fed with 10 μg/L thiamethoxam-contaminated syrup (about 0.25 ng/bee/day); *iv*. Bees fed with 200 μg/L thiamethoxam-contaminated syrup (about 5.0 ng/bee/day); *v*. Bees co-exposed to both CBPV-infected bees and 0.25 ng/bee/day of thiamethoxam; and *vi*. Bees co-exposed to both CBPV-infected bees and 5.0 ng/bee/day of thiamethoxam.

Feeders were changed and weighed and survival was monitored daily (unmarked dead bees were removed from the cages). The volume consumed per bee was estimated taking into account the number of surviving honey bees per cage. At days 5 and 10 post-exposure, 4 whole cages were sacrificed by dipping each cage in liquid nitrogen, and 8 bees (two bees randomly selected from four cages) per condition were sampled then stored at -80°C for analysis of gene expression levels and viral loads.

### Experiment 2: Influence of thiamethoxam and CBPV co-exposure on pesticide metabolisation

Nine-day-old bees were exposed or co-exposed to CBPV and/or thiamethoxam in the same way as previously described but in the following conditions: *i*. Control bees; *ii*. Bees in contact with CBPV-infected bees (nine CBPV-infected bees as inoculum per cage); *iii*. Bees fed 0.25 ng/bee/day of thiamethoxam; and *iv*. Bees co-exposed to both CBPV-infected bees and 0.25 ng/bee/day of thiamethoxam. Similarly, feeders were changed and weighed daily and survival monitored. After 1, 5, 10, 12, 15, and 18 days post-exposure, all the bees from each condition were sacrificed to analyse pesticide residues over time: bees from one cage for Control and CBPV conditions, and bees from three cages for the 0.25 ng/bee/day of thiamethoxam and the 0.25 ng/bee/day of thiamethoxam/CBPV conditions. At day 18 post-exposure, bees from three additional cages were sacrificed for each condition with pesticide exposure. These bees were anesthetised using CO_2_ gas, and then dissected to remove their rectum, where the pesticide residues might accumulate [48]. Samples were stored at -20°C until chemical analysis.

### Quantification of thiamethoxam and clothianidin

Neonicotinoid residues in samples (pools of 20 bees) were quantified using liquid chromatography with electrospray tandem mass spectrometry (LC-MS/MS), according to the protocol described by Martel *et al*. [49]. Briefly, the pesticides were extracted using acetonitrile and liquid partitioning with *n*-hexane. One clean-up was then performed on a florisil cartridge (1 g, 6 mL) and the extract was analysed by LC-MS/MS.

### Quantification of virus and gene expression levels

Viral RNA was extracted from eight individual bees per condition (two bees randomly selected per cage) and CBPV loads were measured in each individual honey bee by quantitative PCR following the protocol described by Schurr *et al*. [50]. The viral loads were expressed in log_10_ of genome equivalent per bee (copies/bee).

Eight additional honey bees were randomly selected from each experimental condition, and total RNA was isolated from each individual bee using TRIzol reagent (Invitrogen, USA) according to the manufacturer’s instructions. The concentration and purity of total RNA were assessed by spectrophotometry (Varioskan Flash Spectral Scanning Multimode Reader; Thermo Fisher Scientific, USA) before being adjusted to the final concentration of 500 ng/μL of RNA.

The expression levels of immune genes (*vitellogenin*, *dorsal-1-a*, *apidaecin*, *ppo*) and detoxication genes (*gst3*, *catalase* and *cyp6as14*) were assessed using a StepOneReal-Time PCR System (Life Technologies, USA) based on a SYBR green detection method. The cycle threshold values of selected genes were normalised to the geometric mean of the housekeeping genes *β-actin* and *rpL32*. Relative gene transcription data were analysed using the 2^ΔΔCt^ method. To verify that the amplification efficiencies of the target and reference genes (*β-actin* and *rpL32*) [51] were approximately equal, amplifications of five 10-fold dilutions of the total RNA sample (from 1,000 to 0.1 ng per reaction) were analysed in triplicate. The efficiency plot for Log input total RNA *vs*. ΔCt curve had a slope lower than ± 0.1, suggesting there was no excessive fluctuations in the transcription of the reference genes.

Amplifications were performed using the Power SYBR Green RNA-to-Ct 1-Step Kit (Thermo Fisher Scientific, USA) with the following thermal cycling profiles: one cycle at 48°C for 15 min for reverse transcription, one cycle at 95°C for 10 min, 40 cycles at 95°C for 15s and 60°C for 1 min, and one cycle at 68°C for 7 min. All primer pairs were designed using PrimerExpress 3.0 software (Life Technologies, USA) following the standard procedure (S1 Table). Negative (H_2_O) and positive controls (previously identified positive samples) were included in each qRT-PCR run.

### Statistics

Survival was established using a Kaplan-Meier estimation [52,53], and curves compared with log-rank tests [54]. Synergistic interactions were tested using a χ^2^ of compliance test comparing survival measurements obtained for each day with the corresponding calculated expected measurements [55,56]. Log_10_-transformed viral loads were analysed using a one-way ANOVA test followed by Tukey HSD tests [57] or Kruskal-Wallis tests followed by Wilcoxon pairwise tests with Bonferroni correction if data were not normally distributed (significant Shapiro-Wilk test). For gene expression analysis, the fold change in ΔCt was calculated using the 2^ΔΔCt^ method using control conditions as the basic reference. Transcription differences were compared using ANOVAs followed by Fisher’s LSD post-hocs or Kruskal-Wallis tests followed by Wilcoxon pairwise tests with Bonferroni correction if data were not normally distributed (significant Shapiro-Wilk test). Differences were considered significant at *p*<0.05. Effect size (Cohen’s *d*) was calculated according to Cohen [58] and using the R software “effsize” package. Effect sizes were considered “small” for *d*<0.5; “medium” for 0.5<*d*<0.8; and “large” for *d*>0.8. Statistical analysis were performed using the software R (Version 3.3.3–2017, Rstudio).

## Results

### Experiment 1: Influence of thiamethoxam and CBPV co-exposure on bee survival, virus loads and physiology

**Fig 1:**
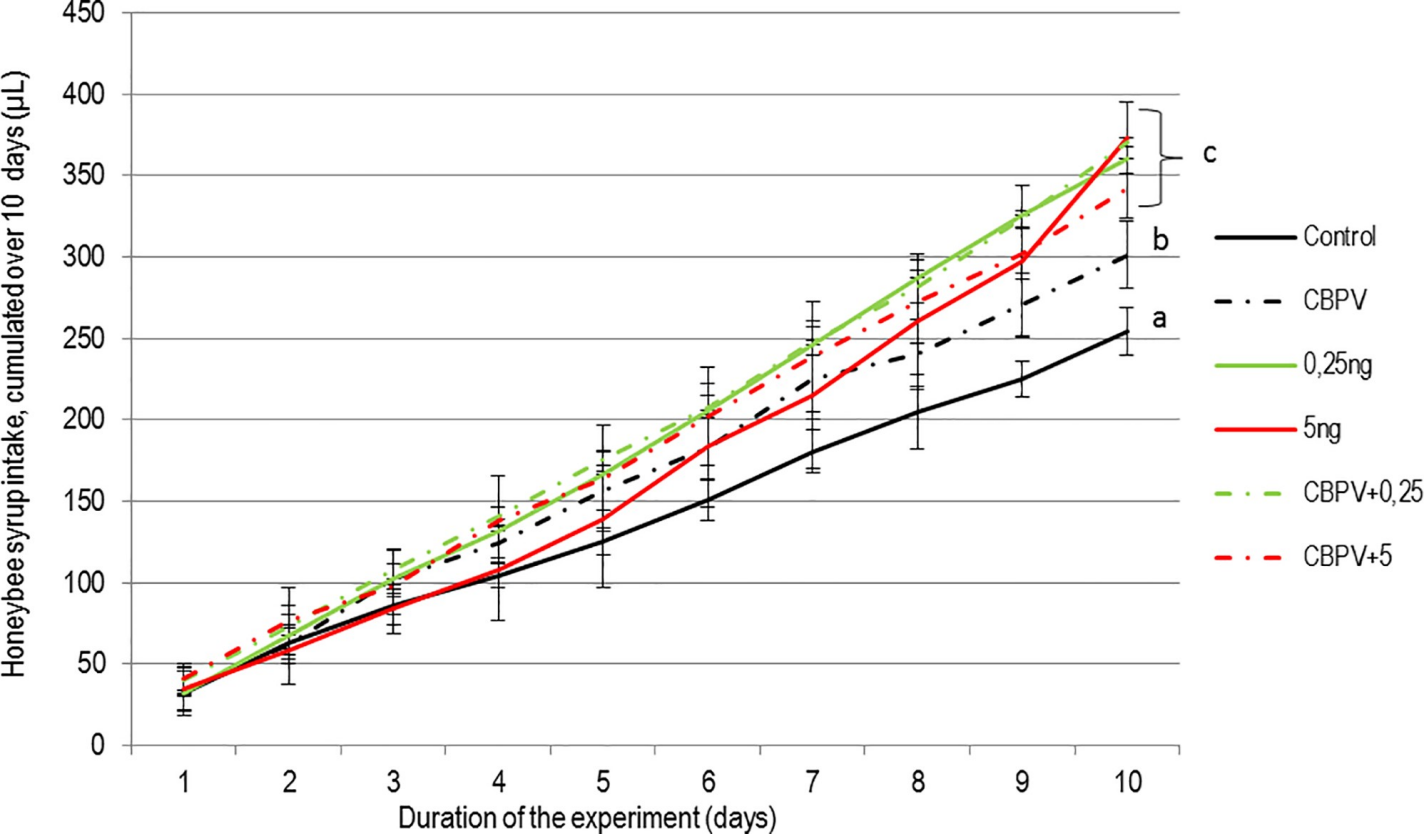
Cumulated syrup intake (μL/bee) of bees exposed to CBPV and/or thiamethoxam. Syrup consumption is shown for each condition: Control bees, CBPV-exposed bees, thiamethoxam-exposed bees (0.25 or 5.0 ng per bee), and bees co-exposed to CBPV and thiamethoxam (0.25 or 5.0 ng per bee). Means and standard deviations of cumulated intakes are shown (syrup intake is normalised considering honey bee survival in the sampled cages). The letters indicate significant differences (p<0.05) found at Day 10 post-exposure.

CBPV infected bees consumed slightly more sugar syrup than control bees over 10 days (at the limit of significance, *p* = 0.05; Fig 1). However, a significant increase in syrup intake was observed in bees exposed to thiamethoxam (with or without CBPV) and regardless of the dose (0.25 or 5.0 ng) (*p*<0.01 for each condition; Fig 1).

#### Survival

**Fig 2:**
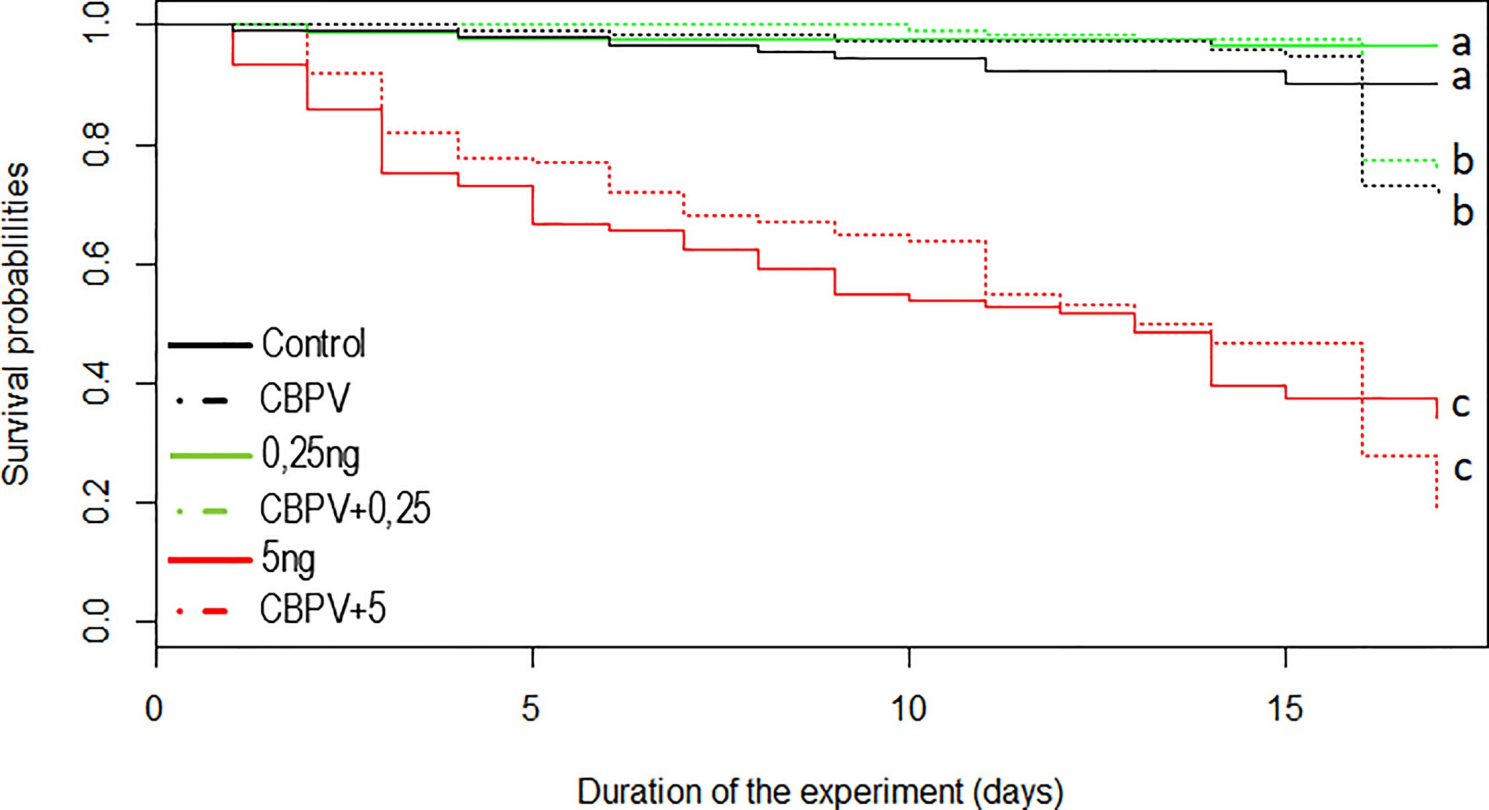
Survival of bees exposed to CBPV and/or thiamethoxam. The survival rate is shown for each condition: Control bees, CBPV-exposed bees, Thiamethoxam-exposed bees (0.25 or 5.0 ng per bee), and bees co-exposed to CBPV and Thiamethoxam (0.25 or 5.0 ng per bee). Letters show statistical differences between conditions (p<0.05). Three significantly different groups emerged from the statistical analysis: a) control bees and bees exposed to 0.25 ng/bee/day of thiamethoxam, b) bees exposed to CBPV alone or co-exposed to 0.25 ng/bee/day of thiamethoxam and CBPV, c) bees exposed to 5.0 ng/bee/day of thiamethoxam or co-exposed to 5.0 ng/bee/day of thiamethoxam and CBPV.

The statistical analysis of the survival rates separated experimental conditions into three different groups (Fig 2). The survival rate of bees exposed to the lowest dose of thiamethoxam (0.25 ng/bee/day) was not different from control bees (*p* = 0.09; *d* = 0,6 (medium); Fig 2). However, bees from both groups exhibited a better survival rate than CBPV-infected bees (*p*<0.01 for both conditions, *d* = 2,4 (large) for Control and 6,5 (large) for bees exposed to 0.25 ng of thiamethoxam) and bees co-exposed to CBPV and 0.25 ng/bee/day of thiamethoxam (*p*<0.01 for both conditions, *d* = 2,04 (large) for Control and 6,5 (large) for bees exposed to 0.25 ng of thiamethoxam). Finally, bees exposed to the highest dose of thiamethoxam (with or without CBPV) exhibited the lowest survival rate (*p*<0.01 when compared to the other conditions, and all *d* were superior to 2 (large effect)).

The survival rates found in the co-exposure conditions did not differ from the mortalities expected from an additive effect of CBPV and thiamethoxam exposure (for both doses; *p*>0.05; *d*<0.5 (small)).

Clinical signs of CBPV infection (trembling bees) have been sporadically observed at the end of the experiment but were not quantified.

#### Viral loads

**Fig 3:**
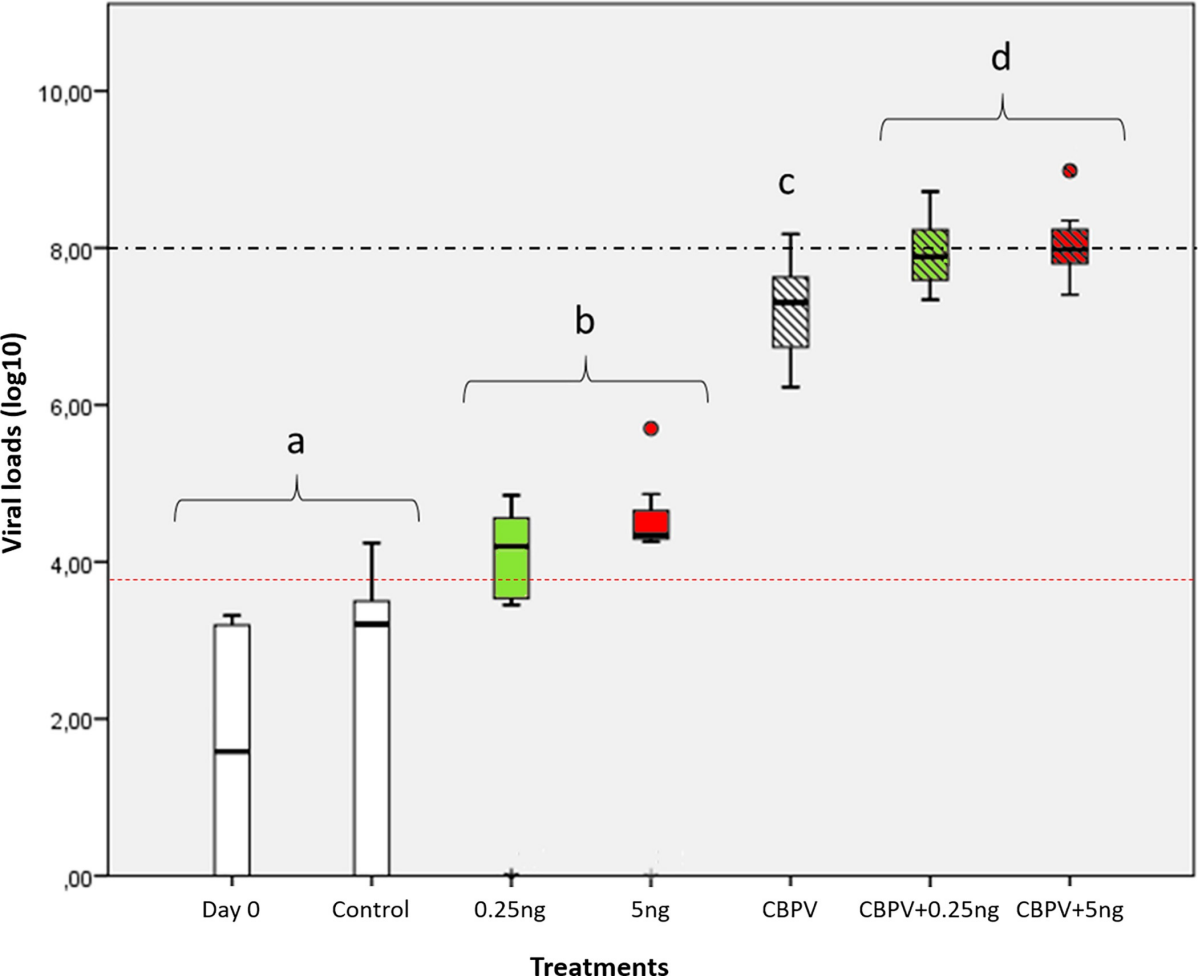
Viral loads (log_10_ of the number of copies/bee) in bees exposed to CBPV and/or thiamethoxam. Day 0 corresponds to newly emerged bees, whereas other measures were performed after 10 days of exposure. The CBPV infection level is shown for each condition: Control bees, CBPV-exposed bees, thiamethoxam-exposed bees (0.25 or 5.0 ng per bee), and bees co-exposed to CBPV and thiamethoxam (0.25 or 5.0 ng per bee) (n = 8 bees per condition). The dashed-dotted black line represents the “infection threshold” (10^8^ copies/individual) above which infected honey bees are known to develop clinical signs of CBPV disease [29]. Different letters show statistical differences between experimental conditions (p<0.05). Box-plots show the distribution of populations, with first quartile (25%), median (50%), and third quartile (75%) (boxes), minimum and maximum (whiskers) and outliers (circles). The dotted red line shows the limit of quantification of the method [29].

As expected, CBPV levels in control bees were low (mostly under the detection threshold) and not significantly different from the levels observed in newly-emerged bees (*p* = 0.38, *d* = 0,6 (medium) Fig 3). The CBPV levels of both groups (Day 0 and Control) were significantly lower than those encountered in bees exposed to both doses of thiamethoxam (*p*<0.03 for both groups; *d*>0,7 (medium) concerning bees exposed to 5.0 ng of thiamethoxam and *d*>1 (large) for bees exposed to 0.25 ng of thiamethoxam). Exposure to CBPV-infected bees induced a significant increase of CPBV in nestmate bees, when compared to control bees (*p*<0.01; *d* = 1,2 (large)), as well as bees exposed to thiamethoxam (*p*<0.02 for both doses, *d =* 1,2 (large) for both doses).

Finally, bees co-exposed to the virus and pesticide exhibited the highest viral loads, which were significantly different from all the other conditions (*p*<0.05, *d*≥0,8 (large) for all tests). In addition, for these co-exposure conditions, the CBPV levels at 0.25 and 5.0 ng of thiamethoxam reached a mean of 1.42 x 10^8^ and 2.08 x 10^8^ copies/bee, respectively, which was above the threshold known for leading to clinical signs of CBPV disease [29].

#### Expression level of immune and detoxication genes

**Fig 4:**
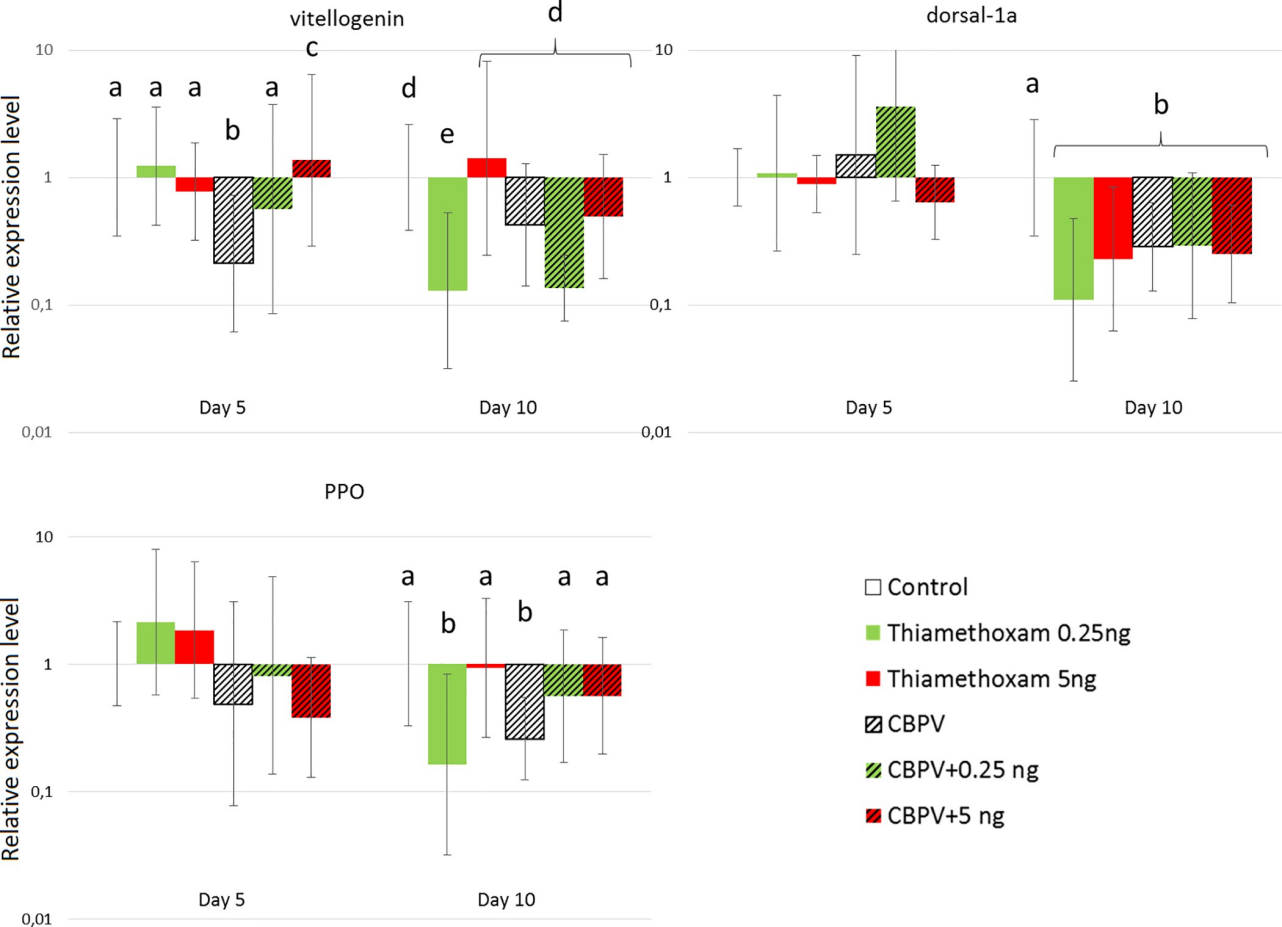
Fold changes in the expression level of immunity-related genes. Eight bees were randomly sampled from each condition. Data were built using the 2^-ΔΔCt^ method, and shown for day 5 and day 10 post-exposure. Letters show statistically different groups.

**Fig 5:**
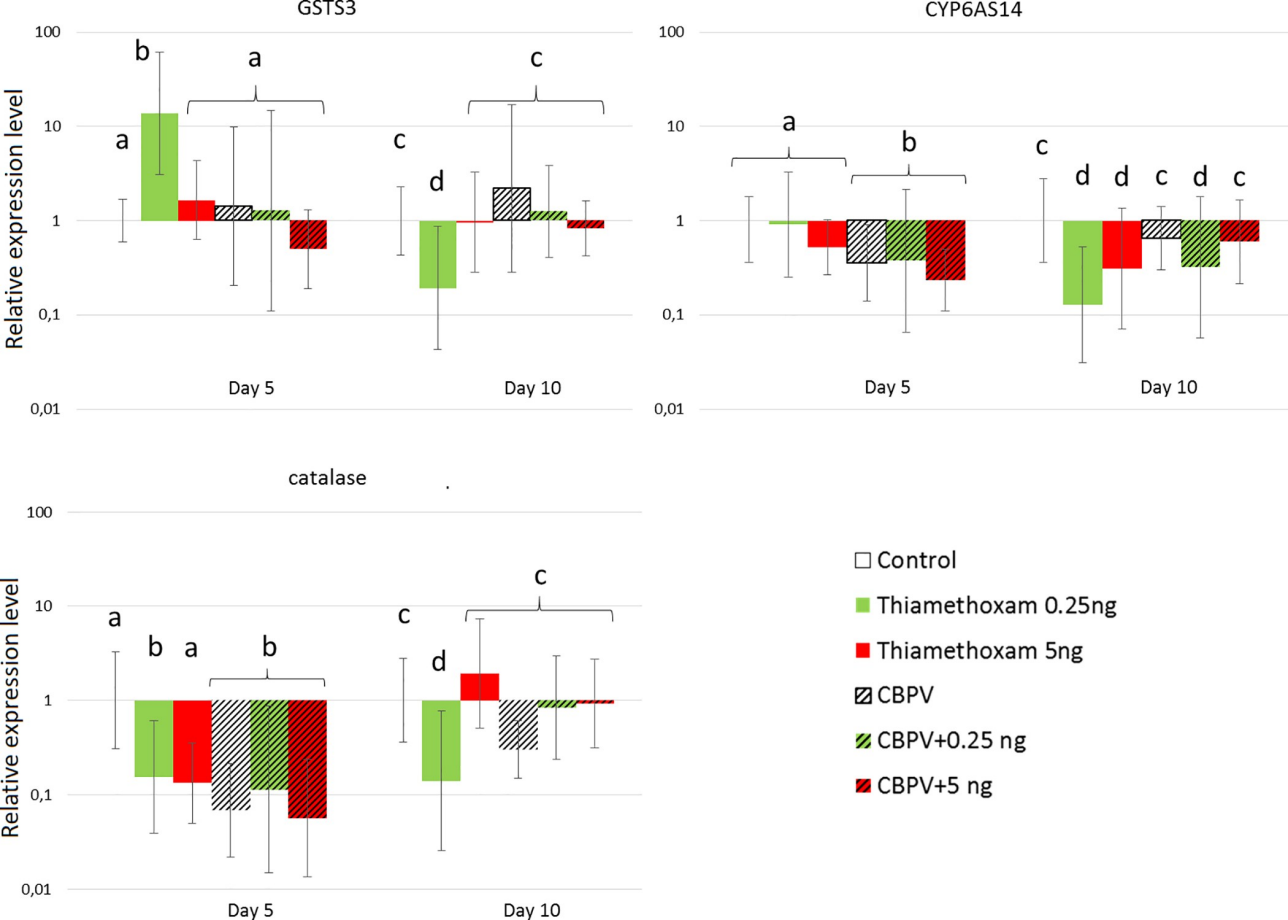
Fold changes in the expression level of detoxication-related genes. Eight bees were randomly sampled from each condition. Data were built using the 2^-ΔΔCt^ method, and shown for day 5 and day 10 post-exposure. Letters show statistically different groups.

The relative changes (compared to the control bees) in transcription of the six selected genes, three immune-related (Fig 4) and three detoxication-related (Fig 5), were measured in honey bees after 5 and 10 days of exposure to thiamethoxam and/or CBPV. Only the transcription level of apidaecin showed no significant differences (*p*>0.05) in any of the conditions (data not shown).

There was no significant effect of exposure to thiamethoxam alone on immunity related genes after 5 days (Fig 4). After 10 days, *vitellogenin*, *dorsal-1a*, and *ppo*, were significantly downregulated by the 0,25 ng exposure (*p*<0.01 for each gene; *d*>5 (large) for all). Bees exposed to the 5.0 ng/bee/day dose of thiamethoxam had significantly lower levels of *dorsal-1a* only (*p*<0.01; *d* = 3.5 (large)). Looking at the effect of thiamethoxam alone on detoxication-related genes, at day 5 (Fig 5), the transcription of the detoxication gene *gsts3* was significantly up-regulated by thiamethoxam at the dose of 0.25 ng/bee/day as compared to control bees (*p* = 0.04; *d* = 2.1 (large)). *Catalase* was down regulated (*p =* 0.044; *d* = 0,95 (large)). At day 10, *gsts3*, *cyp6as14* and *catalase* were significantly downregulated by this same 0.25 ng exposure (*p*<0.01 for each gene, excepted for *catalase p*<0.05; *d*>3,5 (large) for all). Bees exposed to the 5.0 ng/bee/day dose of thiamethoxam had significantly lower levels of *cyp6as14* at day 10 (*p* = 0.01; *d* = 0.93 (large)).

Compared to control conditions, CBPV-infected only bees showed reduced transcription of *vitellogenin* (*p* = 0.01; *d* = 2.2 (large)) 5 days of virus exposure (Fig 4). Transcription of *dorsal-1a* (*p*<0.01. *d =* 0.74 (medium)) and *ppo* (*p* = 0.02; *d =* 2.3 (large)) was reduced after 10 days. CBPV-infected bees had lower levels of *catalase* and *cyp6as14* than control bees at day 5 (respectively *p*<0.01; *d* = 3.6 (large), and *p* = 0.03; *d* = 3.4 (large), respectively) (Fig 5).

Co-exposure of honey bees to 0.25 ng/bee/day of thiamethoxam and CBPV down-regulated *dorsal-1a* after 10 days (*p*<0.01; *d =* 0.88 (large)) (Fig 4), compared to control conditions. Co-exposure to 5.0 ng/bee/day of thiamethoxam and CBPV up-regulated the transcription of *vitellogenin* (*p*<0.01; *d =* 3.9 (large)) after 5 days; and *dorsal-1a* after 10 days (*p*<0.01; *d =* 0.92 (large)). Compared to exposure to the pesticide alone, this co-exposure also significantly up-regulated transcription of *vitellogenin* compared to thiamethoxam at 5 ng alone (*p*<0.01; *d =* 1.7 (large)) at day 10, at day 5 for 0.25 ng (*p* = 0.03; *d =* 2.4 (large)). Finally, if we look at the detoxication-related genes, co-exposure of honey bees to 0.25 ng/bee/day of thiamethoxam and CBPV down-regulated *catalase* after 5 days of exposure (*p*<0.01; *d =* 4.2 (large)) (Fig 5), and *cyp6as14* (p<0.01; *d =* 8.7 (large)) compared to control conditions. After 10 days, it down-regulated *cyp6as14* only (p<0.01; *d =* 1.8 (large)). Co-exposure to 5.0 ng/bee/day of thiamethoxam and CBPV down-regulated the transcription of *catalase* (*p* = 0.01; *d =* 5.3 (large)), and *cyp6as14* (p<0.01; *d =* 2.9 (large)) after 5 days.

### Experiment 2: Influence of thiamethoxam and CBPV co-exposure on pesticide metabolisation

#### Thiamethoxam metabolisation into clothianidin

Control bees as well as thiamethoxam-exposed bees (with or without CBPV co-exposure) did not exhibit clinical signs of overt infection.

**Fig 6:**
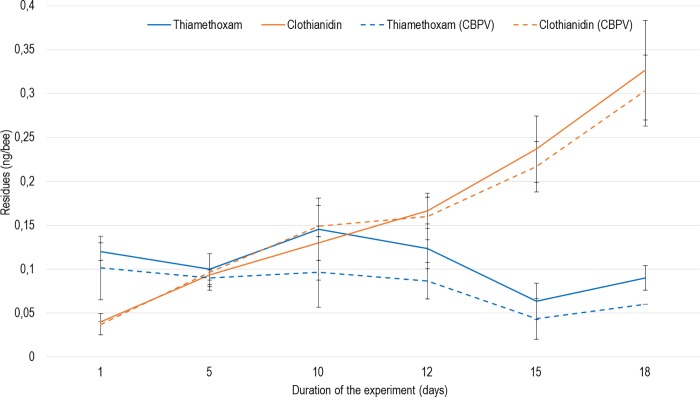
Metabolisation kinetics of thiamethoxam in bees co-exposed or not to CBPV. The metabolisation of thiamethoxam into clothianidin is shown over time in bees exposed to thiamethoxam (0.25 ng/bee/day) and infected or not with CBPV (n = 3x20 bees per condition). Means and standard deviations are shown.

The level of thiamethoxam and clothianidin were under the limit of detection (LOD = 0.015 ng/bee) in control and CBPV-exposed bees over the course of the experiment. In bees exposed to 0.25 ng/bee/day of thiamethoxam, the pesticide level remained stable over the course of the experiment and under 0.15 ng/bee. In contrast, clothianidin levels increased steadily throughout the experiment, from under 0.05 ng/bee after one day of exposure to almost 0.35 ng/bee after 18 days (Fig 6). No significant difference was found at any time and for both pesticides between bees exposed to thiamethoxam and bees co-exposed to thiamethoxam and CBPV.

#### Pesticide residue levels in whole bees and bees devoid of rectum

**Fig 7:**
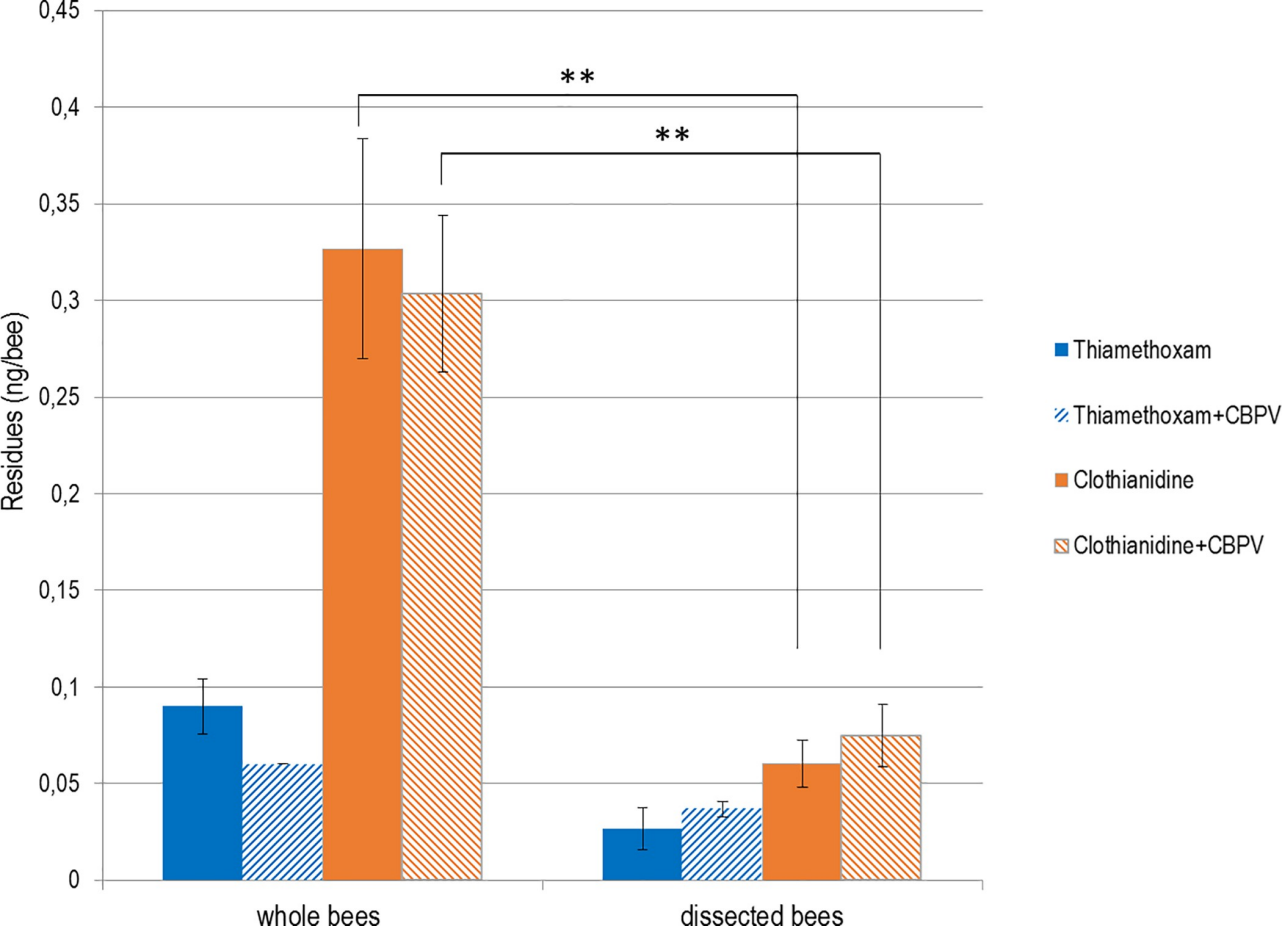
Thiamethoxam and clothianidin levels in whole bees and bees devoid of rectum (n = 20 bees) after 18 days of exposure to 0.25 ng/day/bee thiamethoxam with or without CBPV. Thiamethoxam and its metabolite (clothianidin) levels are shown in whole bees and dissected bees (rectum excised); (**) denotes a significant difference (p<0.01) between the clothianidin measurements in whole and dissected bees. Means and standard deviations are shown.

Thiamethoxam levels between whole bees and dissected bees (rectum excised) did not differ significantly between groups exposed to thiamethoxam and infected or not with CBPV (*p =* 0.35 and *p* = 0.45, respectively; Fig 7). Clothianidin levels were significantly higher in whole bees than in dissected bees, for both treatments (*p*<0.01). However, there was no significant difference in thiamethoxam and clothianidin levels between treatments, either in whole (*p* = 0.33 and *p* = 0.59, respectively) or dissected honey bees (*p* = 0.19 and *p* = 0.28, respectively).

## Discussion

CBPV in bees cause silent or covert infections until they progress to levels associated with more obvious pathological symptoms [29,30]. The mechanisms underlying this transition are not well known. In this study, we found that, when exposed to thiamethoxam, honey bees infected with CBPV levels mimicking covert infection can develop viral loads known to be related to clinical signs of CBPV disease. Moreover, exposure to both stressors significantly reduced the transcription of the immunity gene *dorsal-1a*, which could explain this development of CBPV loads.

We also observed a significant effect of thiamethoxam on syrup intake (Fig 1), which could be explained by the honey bees attraction for the pesticide, making them drink more of it [59]. We observed the same phenomenon in a previous experiment [48]. This phenomenon could contribute to higher exposure levels to the pesticide than previously estimated. A similar effect was observed in CBPV-infected bees alone, but to a lesser extent. In this case, the increase in syrup consumption could be explained by a higher sugar requirement due to the energy invested in fighting the infection [60,61]. Such energetic needs could also be involved in detoxication processes [62]. The co-exposed bees did not exhibit a higher syrup consumption than bees exposed solely to thiamethoxam, underlining the absence of interaction between the virus and the pesticide on this physiological trait.

We observed that CBPV viral loads in the tested honey bees increased gradually with the presence of pesticide and at any dose, after exposure to CBPV-infected bees (Fig 3). The CBPV levels in virus-pesticide co-exposed honey bees were the only ones to exceed the number of viral copies per bee that is usually related to the development of clinical signs (10^8^ CBPV genome copies per bee)[29]. Therefore, high CBPV levels in the co-exposure conditions could result from an interaction between thiamethoxam and CBPV, regardless of the dose. However, despite the significant increase in CBPV levels, we did not observe a synergistic effect of the virus/pesticide co-exposure on short-term bee mortality 15 days after exposure (Fig 2). Compared to our previously reported results [48], we found a relatively low impact of CBPV and of thiamethoxam on survival. This better survival in the present study could be explained by the use of winter bees instead of summer bees. We used bees from a winter apiary in order to minimize viral contamination from the natural environment, notably by pollen, and colony drifting [63,64]. Exposed to natural resources and other colonies, there is no way to obtain uncontaminated honey bees, especially in summer. The summer honey bees used in our previous experiment (45) had been selected from the colonies with the lowest viral loads in our apiary. However, they were still contaminated with about 10^5^ copies per bees of SBV, and between 10^4^ and 10^6^ copies per bees of BQCV. Nevertheless, these viral loads were considered low, close to the limit of detection of the PCR methods[30]. This difference in the initial viral loads might not solely explain the differences between both experiments, neither the influence of SBV and BQCV, since they were found to have effects on brood only [14,22]. To ensure the new experiment wouldn’t carry those biases, and because the food intake and drifting could not be controlled during foraging season, we selected the winter period for this new study. Our colonies were thus kept in the winter apiary and were fed with artificial food. Emerging bees were tested for viruses and we did not detect any SBV, BQCV, ABPV nor DWV. Winter weather keeping bees inside also decreases the risk of horizontal contamination through drifting and/or robbing of foragers between colonies [64]. Consequently, the viral status of the honey bees used for the experiments was improved, with bees withviral loads below the limit of detection, but physiological differences cannot be ruled out between our bees and summer bees. Winter bees notably have larger fat bodies and possess more phenoloxidase, and have their vitellogenin rates decline more slowly than summer bees [65–68]; both proteins are known to support a stronger immune system when at high levels [69]. Moreover, winter honey bees have been previously found to be less sensitive to pesticides than summer honey bees [70–72]. Further experiments comparing for example *vitellogenin* gene transcription levels and fat body size between summer-born, winter-born (the honey bees used in this study) and true winter honey bees could help confirm or not this hypothesis. Indeed, our honey bees might not be true winter bees, as they were born during winter and not before it. Differences between the two experiments may also arise from differences in bee genetic background [73–75], as different colonies have been used.

In order to explain the mechanisms underlying the increase in CBPV levels after thiamethoxam exposure, we studied the expression of several genes involved in immunity and detoxication (Figs 4 and 5). Exposure of honey bees to CBPV alone caused a down-regulation of immune-related genes, which is contrary to what would be expected in response to a pathogenic infection (Fig 4). However, a similar effect on *vitellogenin* gene transcription has been observed in previous studies, caused by various stresses, including but not limited to viruses [44,76–78]. Other viruses, such as DWV, have been found to induce a down-regulation of *dorsal-1a* [79]. Nevertheless, we found that the transcription of the tested genes, after five days of co-exposure to thiamethoxam at 5.0 ng/bee/day and CBPV, were statistically not different from the observed transcription of the same genes in bees exposed CBPV alone and to the pesticides alone. After ten days, however, the responses after co-exposure corresponded only to the response found after exposure to the pesticide alone. Indeed it has been shown that, as bees age, some immune response gene transcriptions fade, and sometimes even stop completely [80,81], which could explain this absence of reaction to the infection.

Our experiments performed with honey bees exposed only to thiamethoxam did not show dose-dependent variations in gene transcription, but different, sometimes opposite variations (Fig 4). For example, the lowest dose of 0.25 ng/bee/day had a significant down-regulating effect on all tested genes after 10 days of exposure, when 5.0 ng/bee/day had this effect only on a few (*dorsal-1a* and *cyp6as14* genes). This differential response between two very different doses could be explained by a phenomenon known as hormesis. Hormesis can be defined as a biphasic dose-response whereby exposure to low doses of a stressor can stimulate biological processes more efficiently than high doses [82].

We also found that thiamethoxam down-regulated *dorsal-1a* and *catalase*. The down-regulation of *dorsal-1a* is in accordance, while happening later, with the results of Di Prisco *et*. *al* [15] on clothianidin. Indeed, thiamethoxam toxicity is slightly lower than clothianidin [83,84]. The thiamethoxam 48 h oral lethal dose (LD50) has been estimated to vary between 1.65 ng/bee and 9.07 ng/bee depending on the European subspecies [73]. Here, even though metabolisation of thiamethoxam to clothianidin is high (Fig 6), we showed that most of the resulting clothianidin is excreted in the rectum (Fig 7); then, is not supposed to be toxic for the bee [85]. This down-regulation of *dorsal-1a* could explain the higher CBPV loads in honey bees exposed to the pesticides alone. Since bees were not free of CBPV before the experiment, the down-regulation of this immune-related gene would have allowed for this covert infection to develop into higher viral loads. For another virus, Nazzi et al. [79] showed that DWV down-regulated *dorsal-1a*, affecting the NF-κB effector, thus allowing DWV to replicate uncontrollably and reach high infection levels. Here, we hypothesise that a similar effect might underly the significant increase in background CBPV infection in bees co-exposed to thiamethoxam. In addition, the down-regulation of *catalase* by both stressors (pesticide and virus) is unexpected (Fig 5). One of the roles of catalase is to protect cells from a dangerous and ubiquitous metabolic byproduct, H_2_O_2_ [86]. Indeed, in addition to being a byproduct of xenobiotic metabolisation [86], H_2_O_2_ is known to be produced as an innate response to viral infections in most vertebrates, and insects [87,88]. Nevertheless, we found that the down-regulation of the detoxication-related *catalase* and *cyp6as14* genes by co-exposure, has no impact on the metabolisation kinetics of thiamethoxam. This underlines the fact that, under these experimental conditions, the detoxication system seems relatively more robust than the immune system. The metabolisation of thiamethoxam could be performed by different enzymes or pathways of the bee midgut [85] that we have not tested; indeed, the down-regulation of *cyp6as14* here suggests that this enzyme has no an impact on the metabolisation process. CYP450, for example, is a large family of detoxication-related enzymes, of which a number are present in the honey bee [89]. Further studies are needed to uncover specifically which detoxication pathways or enzymes play a key role in the detoxication of this specific neonicotinoid in bees. For instance, a specific P450 enzyme (*cyp9q3)* has been found to give bees their relative insensitivity to thiacloprid as compared to imidacloprid [90].

Our results, while interpretable, are also not totally concurring with the literature, and reflect the difficulty of working on a few single selected genes. More bees might also be needed to avoid bias brought by inevitable individual variations. In the future, better-suited tools such as RNAseq should be used to uncover the potential gene expression changes in bees exposed or co-exposed to both stressors.

In conclusion, the co-exposure to CBPV and thiamethoxam of honey bees from a winter apiary had less effect than the previous reported effect on summer honey bees [48]. However, by using bees with very low initial loads, we showed that low doses of thiamethoxam could trigger chronic bee paralysis replication in honey bees reaching 10^8^ copies/bees, documented as a threshold where bees start developing symptoms of overt infection [29]. Nevertheless, due to a specific physiological state, winter bees are usually more tolerant to a variety of stressors, therefore complementary experiments on this co-exposure should be considered on summer bees, taking into account the compromising between the use of honey bees with the lowest possible viral loads and environmental relevance. Notably, overt infections with CBPV and thiamethoxam exposure are much more likely to occur in spring and early summer; even if contamination of wax and stored food is to take into account, as it was found to be a frequent occurrence [91–95].

## Supporting information

S1 Table Primers used for the quantification of selected honey bee genes and CBPV. (DOCX)
