# Supplementary material for: Influence of chronic exposure to thiamethoxam and chronic bee paralysis virus on winter honey bees
Source: PLoS One. 2019 Aug 15;14(8):e0220703. doi: 10.1371/journal.pone.0220703 (PMC6695216; doi:10.1371/journal.pone.0220703)
Supplement: S1 Table — (DOCX) [file pone.0220703.s001.docx]

# Supplementary material

| **Name** | **Sequence (5’-3’)** | **Amplicon length (bp)** | **Reference** |
| --- | --- | --- | --- |
| *CYP6AS14* |  |  |  |
| F | TGACATTGAGTTGACGGACGAT | 64 | this study |
| R | GAAACCTGCCGCGAAGAA |  |  |
| GSTS3 |  |  |  |
| F | AAACCGATAGCGCAGAGTAACG | 87 | this study |
| R | CATCATTGCCTCCCATTCGT |  |  |
| Catalase |  |  |  |
| F | TTTGGTGGGCCTAGAGAATGTC | 92 | this study |
| R | TCCTCCTTTGGGTCTACATCATAAC |  |  |
| Apidaecin |  |  |  |
| F | TTTTGCCTTAGCAATTCTTGTTG | 81 | [8] |
| R | GTAGGTCGAGTAGGCGGATCT |  |  |
| Dorsal-1A |  |  |  |
| F | TCGGATGGTGCTACGAGCGA | 153 | [8] |
| R | AGCATGCTTCTCAGCTTCTGCCT |  |  |
| Vitellogenin |  |  |  |
| F | AACGCCGTGAAGGTGAACAG | 109 | this study |
| R | TATCGTAGAGAACCTCGCATTTCC |  |  |
| PPOAct |  |  |  |
| F | CGTTGAAAAGTCGAAGCAGATTAA | 112 | this study |
| R | AGGACGCCACCGCAGTATT |  |  |
| β-actin |  |  |  |
| F | GATTTGTATGCCAACACTGTCCTT | 69 | [8] |
| R | TTGCATTCTATCTGCGATTCCA |  |  |
| RpL32 |  |  |  |
| F | CGTCATATGTTGCCAACTGGTTT | 107 | this study |
| R | CCATGAGCAATTTCAGCACAA |  |  |
| CBPV |  |  |  |
| qCBPV9 | CGCAAGTACGCCTTGATAAAGAAC |  |  |
| qCBPV10 | ACTACTAGAAACTCGTCGCTTCG | 101 | [29] |
| CBPV S2 | (6-Fam)TCAAGAACGAGACCACCGCCAAGTTC (Tamra) |  |  |

**Supplementary Table 1:** Primers used for the quantification of selected honey bee genes and CBPV.
